# Female sexual dysfunction after breast cancer surgery prediction with AI

**DOI:** 10.1192/j.eurpsy.2021.1612

**Published:** 2021-08-13

**Authors:** A. Mereu

**Affiliations:** Research performed independently, Cagliari, Italy

**Keywords:** Artificial Intelligence, cancer, breast, surgery

## Abstract

**Introduction:**

Female sexual dysfunction (FSD) can be overlooked. Different types of breast cancer surgery could have a different impact on the sexuality of women. Artificial intelligence (AI) could help to determine the relation between those conditions.

**Objectives:**

To investigate whether AI could predict FSD relying primarily on the time elapsed after treatment and the type of breast cancer surgery.

**Methods:**

Data of age, time elapsed after treatment and type of surgery (breast-conserving therapy and mastectomy) were employed to predict FSD status in 128 subjects using an AI. Women with and without steady relations were included in the analysis. FSD prevalence was 27.3%. The AI was conservatively tuned to maximize the positive likelihood ratio considering predicted and real FSD statuses. The free and open source programming language R was used for all the analyses. Dataset source: Nowosielski, Krzysztof; Krzystanek, Marek; Kowalczyk, Robert; Streb, Joanna; Kucharz, Jakub; Głogowska, Iwona; Lew-Starowicz, Zbigniew; Cedrych, Ida (2018), “Data for: Factors affecting sexual function and body image of early stage breast cancer survivors in Poland: A short-term observation.”, Mendeley Data, V1, doi: 10.17632/948n98trm6.1

**Results:**

Predictions obtained a positive likelihood ratio of 5.314. The results were indicative of fair performance.

**Conclusions:**

AI might be useful to predict FSD in women who undergo breast cancer surgery. Furthermore, the results of this study might indicate a moderate effect of age, time after treatment and type of surgery on the probability of FSD occurrence. Finally, the AI used in this study is freely available, allowing anyone to experiment.

